# Is There Association between Vitamin D Concentrations and Body Mass Index Variation in Women Submitted to Y-Roux Surgery?

**DOI:** 10.1155/2018/3251675

**Published:** 2018-05-03

**Authors:** Maria Tereza A. dos Santos, Fabiola Isabel Suano-Souza, Fernando Luiz Affonso Fonseca, Marise Lazaretti-Castro, Roseli Oselka Saccardo Sarni

**Affiliations:** ^1^Faculdade de Medicina do ABC, Santo Andre, SP, Brazil; ^2^Universidade Federal de São Paulo, Escola Paulista de Medicina, São Paulo, SP, Brazil; ^3^Faculdade de Medicina do ABC, Universidade Federal de São Paulo, Campus Diadema, Diadema, SP, Brazil

## Abstract

**Objective:**

To evaluate vitamin D deficiency and body composition of women submitted to bariatric surgery and relate their body mass index variation after surgery to 25(OH)D concentrations.

**Method:**

A cross-sectional and controlled study was performed including 49 obese adult volunteer women, submitted to Roux-en-Y gastric bypass (RYGB group).

**Collected Data:**

Body mass index (BMI), self-declared ethnicity, economic condition, physical activity level, serum concentrations of 25-hydroxycholecalciferol (25(OH)D; radioimmunoassay), parathormone, and body composition by dual-energy X-ray absorptiometry (Hologic DXA-QDR-1000) were collected.

**Results:**

25(OH)D deficiency was found in 27 (55.1%) and 8 (21.1%) in the RYGB and control groups (*p*=0.002). Secondary hyperparathyroidism was more frequent in the RYGB group compared to the control group (15 (30.6%) versus 1 (2.6%); *p*=0.001). There was no relation of the studied variables and body composition with 25(OH)D deficiency. 25(OH)D concentrations were correlated (*r*=−0.531; *p* < 0.001) with BMI reduction, regardless of vitamin D supplementation.

**Conclusion:**

Women submitted to bariatric surgery (RYGB) around three years ago had higher BMI and vitamin D deficiency, along with hyperparathyroidism, compared to the control group. There was no association between variables related to body composition and 25(OH)D concentrations. On the other hand, vitamin concentrations correlated negatively to BMI variation after undergoing surgery.

## 1. Introduction

Obesity, a chronic illness with a complex treatment, is a serious public health issue that affects millions of people all over the world. According to data from the Surveillance System for Risk and Protective Factors for Chronic Diseases by Telephone Survey (Vigitel), conducted by the Brazilian Ministry of Health, including data collected from 26 capital cities and the Federal District in 2014, 52.5% of Brazilians were overweight [1].

Regarding morbid obesity (BMI > 40 kg/m^2^) tendency on populational surveys carried out in Brazil since the 1970s, there was a 255% increase, going from 0.18% (in 1974–1975) to 0.33% (in 1989) and 0.64% (in 2002-2003) [2]. Morbid obesity in Brazilian adults affects approximately 609,000 individuals, who would be possible candidates for bariatric surgery [3].

The number of bariatric surgeries performed in the country in the last 50 years has considerably increased since it is one of the most sustainable weight loss methods for morbidly obese people so far. Between 2001 and 2014, 49,425 bariatric surgeries were performed by the Unified Health System (SUS) [4].

Monitoring the nutritional condition, body composition, and dietary intake of these patients in the short- and long-term is fundamental for planning interventions, health maintenance, and their quality of life. Among the most prevalent nutritional deficiencies is the lack of vitamin D. It is a frequent condition in obese individuals, and some factors may contribute to this association, such as low food consumption, less sun exposure, and retention in the adipose tissue, leading to less bioavailability of vitamin D [5].

A recent publication described the relationship between body composition (subcutaneous and omental cell tissues) and total stocks of 25-hydroxyvitamin D (25(OH)D) in obese individuals submitted to bariatric surgery and healthy controls. Total stocks were higher in the operated obese group, with no difference in serum concentrations of 25(OH)D. The results reinforce the hypothesis of volumetric dilution of vitamin D in the adipose tissue found in obese individuals [6, 7].

Literature points out the importance of micronutrient deficiency, emphasizing vitamin D, in individuals previously submitted to bariatric surgery [8]. However, so far, there have been doubts regarding the ideal doses recommended for supplementation to prevent the deficiency [9]. A systematic review, including 14 studies (*n*=2688 individuals), has shown that hyperparathyroidism associated with vitamin D deficiency persists up to five years after bariatric surgery, regardless of supplementation with calcium and vitamin D [10].

Regarding body composition, longitudinal studies conducted with patients submitted to bariatric surgery have shown a decrease in fat mass and lean mass in approximately 30–35% and 10–15%, respectively. The decrease was especially noticed during the first three to six months after surgery [11, 12].

Previous publication showed that there is a progressive redistribution of fat mass that adopts a metabolically healthy pattern two years after undergoing surgery [13].

The present study was performed considering the relevance of the subject and the knowledge gaps concerning the behavior of biomarkers associated with vitamin D deficiency in relation to body composition of obese women who have undergone bariatric surgery. We aim to evaluate 25(OH)D deficiency and the body composition of women who were subjected to bariatric surgery and relate the variation of BMI after surgery to 25(OH)D concentrations.

## 2. Methods

A cross-sectional and controlled study evaluated 49 adult female volunteers, submitted to RYGB 3.3 ± 1.1 years before (2.0 to 6.0 years) (RYGB group), between 2001 and 2004, in a public and teaching hospital in the city of Santo André, SP. The study excluded patients with chronic diseases (kidney, liver, and rheumatic) and/or those who were receiving medication (anticonvulsant, corticosteroid, antiretroviral, and hormone replacement) that could interfere with vitamin D concentrations. After the surgery, all women were advised daily and continuous use of vitamin and mineral supplements containing 200 UI of vitamin D and 250 mg of calcium.

Forty-one healthy nonobese volunteers with similar age and socioeconomic condition to those of the study group were recruited to compose the control group. The volunteers worked in operational positions in the same public hospital where the patients were operated.

The project was approved by the Research Ethics Committee of Faculdade de Medicina do ABC (FMABC) *n*. 176/2005 and all women signed the consent form to take part in the study.

### 2.1. Clinical Data and Life Habits

The physical activity level was assessed based on the International Physical Activity Questionnaire (IPAQ), short version [14].

### 2.2. Body Composition

The women who had undergone RYGB and the control group were submitted to anthropometric evaluation—weight and height—using an analog scale (0.1 kg scale, Filizola model 31, São Paulo, SP) to classify the nutritional state based on the BMI [15].

Dual-energy X-ray absorptiometry (DXA) was performed with an Hologic equipment, Discovery A model (Waltham, USA), and used for the assessment of body compartments. DXA exams were carried out at the Metabolic Bone Disease Clinic of Federal University of Sao Paulo, Brazil (Universidade Federal de São Paulo-Escola Paulista de Medicina), always using the same technique. Reports were issued by the same professional. In order to analyze body composition, we considered BMI (kg/m2), fat mass (kg), fat percentage (%), total muscle mass (kg), skeletal muscle mass (kg), and lean mass index (lean mass (kg)/height^2^).

### 2.3. Biochemical Evaluation

Blood samples (20 ml) were taken between September and December, in the morning and after 12 hours of fasting. Then, they were stored at 4°C temperature and transported to the Clinical Analysis Laboratory of Faculdade de Medicina do ABC, Santo Andre, Brazil, in no more than 30 minutes, where they were centrifuged for 10 minutes at 10,000 rpm and distributed into aliquots for further laboratory analysis.

The following biomarkers of vitamin D status were analyzed: 25(OH)D (radioimmunoassay method; DiaSorin) and intact parathormone (PTH) (electrochemiluminescence; Elecsys, Roche). To classify 25(OH)D concentrations, the cutoff points suggested by Borradale and Kimlin [16] were adopted.

### 2.4. Statistical Analysis

For statistical analysis, the program used was SPSS 24.0 (IBM®). The categorical variables were presented in absolute and percentage numbers, compared by the chi-square test. The continuous variables were assessed for their normality. Since they displayed normal distribution, they were presented in the form of mean ± standard deviation and compared through Student's *t*-test. The Pearson correlation was used to measure the association between 25(OH)D and BMI variation. Binary logistic regression was used to identify the factors associated with 25(OH)D inadequacy and body composition in the RYGB group. The significance level adopted was 5%.

## 3. Results

In the group of women submitted to RYGB, the average age was 45.0 ± 9.0 years (minimum and maximum: 23 to 63 years) and the time after surgery was 3.3 ± 1.1 years (minimum and maximum: 2.0 to 6.0 years). The average weight loss was 46.9 ± 16.4 kg (minimum and maximum: 9 to 80 kg) and BMI reduction was 30.7 ± 4.7 kg/m^2^ (minimum and maximum: 23.8 to 43.1 kg/m^2^).

The RYGB group, compared to the control group, had more Caucasian women (35 (71.4%) versus 16 (39.0%); *p*=0.002), more frequent use of supplements containing vitamin D (33 (67.3%) versus 1 (2.4%); *p* < 0.001), and lower percentage of smoking (3 (6.1%) versus 12 (29.3%); *p*=0.004) (Table 1).

The average 25(OH)D concentrations in the RYGB and control groups were 22.7 ± 11.8 ng/ml and 28.9 ± 8.9 ng/ml (*p*=0.008), respectively. 25(OH)D values compatible with deficiency (<20 ng/ml) were observed in 27 (55.1%) and 8 (21.1%) in the RYGB and control groups (*p*=0.002). Secondary hyperparathyroidism was more frequent in the RYGB group when compared to the control group (15 (30.6%) versus (2.6%); *p*=0.001).

In Table 2, after binary logistic regression, we verified that 25(OH)D deficiency was more frequent in the RYGB group, even after adjusting for ethnicity, smoking, BMI, and vitamin supplementation.

When comparing variables related to nutritional condition and body composition between RYGB and control groups, multivariate analysis showed that only the highest BMI was independently associated with the RYGB group (*β* = 1.48; 95% CI: 1.01 to 2.19; *p*=0.045) (Tables 3 and 4).

**Figure 1:**
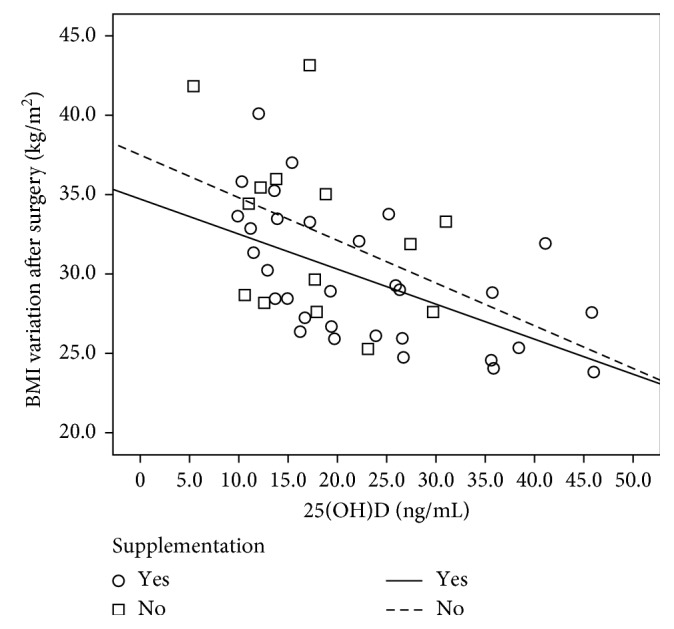
Association between vitamin D concentrations and BMI variation in the group submitted to RYGB with and without supplementation. The Pearson correlation between 25(OH)D and BMI variation (*r*=−0.531; *p* < 0.001).

There was no association between variables related to body composition and 25(OH)D concentrations (Table 5). In turn, 25(OH)D concentrations correlated negatively (*r*=−0.531; *p* < 0.001) with BMI variation after the RYGB; that is, 25(OH)D concentrations were lower in individuals with higher BMI variation, regardless of vitamin D supplementation (Figure 1).

## 4. Discussion

The present study points out to the high percentage of 25(OH)D deficiency (55.1%) and secondary hyperparathyroidism in women submitted to RYGB, three years after the procedure. Women who were previously operated displayed higher BMI compared to those in the control group. There was no association between body composition evaluated by DXA and 25(OH)D concentrations.

A meta-analysis involving 15 articles with 3867 obese individuals and 9342 healthy individuals of Asian and European American origin concluded the association between obesity and vitamin D deficiency [17]. Concerning bariatric surgery, evidence showed that vitamin D deficiency occurs before the surgery and persists for long periods after the procedure [18]. A review study conducted by Chakhtoura et al. emphasizes that, for individuals to reach 25(OH)D concentrations higher than 20 ng/ml in the postoperative period, they required high doses of supplementation, between 1100 and 7100 UI. However, authors point out that there are many methodological limitations in the studies included [19].

A systematic review including 14 studies (*n*=2688) showed a gradual PTH increase along with a progressive reduction in 25(OH)D concentrations at two years, between two and five years, and from five years onwards after RYGB [10]. Another study with obese individuals, considering the periods before surgery and one and four years after laparoscopic sleeve gastroplasty demonstrated vitamin D deficiency during all the supervised periods, with 96.2%, 89%, and 86%, respectively. PTH levels increased progressively [20]. The guideline suggests daily supplementation of 1200 to 1500 mg of calcium and 3000 UI of vitamin D after bariatric surgery [21].

In the present study, vitamin D deficiency was more frequent in the RYGB group, even after adjusting for variables such as ethnicity, smoking, BMI, and vitamin supplementation. Vitamin D deficiency in postbariatric surgery patients has been related to several factors. Some of them are associated with excess adiposity and others with the type of surgical procedure [5]. In this study, there was no observed association between 25(OH)D concentrations and adiposity in women submitted to RYGB. Bioavailability of vitamin D in obese people is reduced, and this is credited to retention by the visceral and subcutaneous adipose tissue. Even if there is normal synthesis, absorption may be compromised due to anatomic alterations in the surgical procedure. It is believed that obese individuals can display reduction in the expression of enzymes involved in the metabolism of vitamin D, 25-hydroxylase, and 1α-hydroxylase present in the visceral and cutaneous adipose tissue [22].

After multivariate analysis, it was noticed in the present study that only BMI, not the other variables of body composition evaluated by DXA, was independently associated with the RYGB group.

Longitudinal studies have shown favorable results regarding the body composition of individuals submitted to bariatric surgery. Bazzocchi et al. described the positive balance between fat mass and lean mass in women submitted to bariatric surgery two years before. The authors showed a lean mass reduction in the first three months, with further stabilization [13]. Similar results were published by Ciangura et al. [23]. The results found in the present study can come from the cross-sectional study design, considering that longitudinal studies demonstrate that body composition changes are dynamic and modify over time.

Another variable influencing body weight loss and metabolic parameters after RYGB is the length of the alimentary (A) and biliopancreatic (BP) limbs. Nergaard et al. in a prospective randomized study found that a 2 m BP limb gives a better long-term weight loss than RYGB with a 60 cm BP limb and a 150 cm A limb. The 2 m BP limb needed significantly more often supplementation adjustment (iron, vitamin D, and calcium) than the other group [24].

In this study, 25(OH)D concentrations correlated negatively to the rate of BMI reduction after the person was submitted to RYGB, regardless of vitamin D supplementation. Literature shows that weight loss resulting from conventional clinical treatment for obesity is followed by an increase in 25(OH)D concentrations [9]. A possible explanation for the inverse association observed would be the lower absorption of nutrients, including vitamin D, in individuals with greater BMI reduction.

## 5. Conclusion

Women submitted to bariatric surgery (RYGB) around three years ago presented higher BMI and vitamin D deficiency, along with hyperparathyroidism, compared to the control group. There was no association between variables related to body composition and 25(OH)D concentrations. On the other hand, vitamin concentrations correlated negatively to BMI variation after undergoing surgery. Future studies evaluating the impact of supplementation on biomarkers associated with vitamin D deficiency and taking into account the length of the alimentary and biliopancreatic limbs are necessary.

## Figures and Tables

**Table 1:** Characterization and comparison of RYGB and control groups (*n*=90).

	RYGB group (*n*=49)	Control group (*n*=41)	*p* value
Age (years)	45.0 ± 9.0	43.7 ± 9.1	0.232^1^
Ethnicity (white)	35 (71.4%)	16 (39.0%)	0.002^2^
Menopause (yes)	19 (38.7%)	16 (39.0%)	0.576^2^
Use of OCP (yes)	6 (12.2%)	7 (17.1%)	0.559^2^
Supplementation (yes)	33 (67.3%)	1 (2.4%)	<0.001^2^
Regular photoprotection (yes)	20 (40.8%)	23 (56.1%)	0.204^2^
Physical activity (yes)	31 (63.3%)	29 (70.7%)	0.506^2^
Smoking (yes)	3 (6.1%)	12 (29.3%)	0.004^2^
25(OH)D <20 ng/dL	27 (55.1%)	8 (21.8%)	0.009^1^

OCP: oral contraceptive pill; ^1^significance level of Student's *t*-test; ^2^significance level of the chi-square test.

**Table 2:** Multivariate logistic analysis of 25(OH)D concentrations in the RYGB group (*n*=49).

Variable	B	*β*	95% CI		*p* value
Ethnicity (white)	1.73	5.65	0.96	33.14	0.055
Vitamin supplementation (yes)	5.45	232.9	12.17	459.45	<0.001
Smoking (yes)	−2.34	0.096	0.01	1.42	0.089
BMI > 25 kg/m^2^	2.49	12.08	12.08	0.78	0.075
25(OH)D < 20 ng/ml	2.29	9.92	1.63	60.4	0.013

BMI: body mass index; dependent variable: 25(OH)D.

**Table 3:** Body composition of the group of women submitted to RYGB and control group (*n*=90).

Variable	RYGB group (*n*=45)	Control group (*n*=38)	*p* value
Weight (kg)	79.5 ± 11.3	65.3 ± 9.4	<0.001
BMI (kg/m^2^)	31.7 ± 4.4	26.3 ± 3.1	<0.001
Total muscle mass (kg)	47.4 ± 5.1	41.0 ± 4.7	<0.001
Total skeletal muscle mass (kg)	17.9 ± 5.8	14.9 ± 4.9	0.018
Skeletal muscle mass index (kg/m^2^)	7.6 ± 1.2	6.5 ± 0.7	<0.001
Fat mass (kg)	30.0 ± 8.2	22.6 ± 5.8	<0.001
Total fat percentage (%)	37.0 ± 6.0	34.0 ± 4.2	0.015

BMI: body mass index; significance level of Student's *t*-test.

**Table 4:** Multivariate logistic analysis of body composition of RYGB and control groups (*n*=90).

Variable	B	*β*	95% CI		*p* value
Body mass index (kg/m^2^)	0.397	1.487	1.01	2.19	0.045
Lean mass (kg)	−0.439	0.645	0.25	1.67	0.368
Adipose mass (kg)	0.749	2.114	0.33	13.3	0.426
Fat (%)	−0.437	2.22	0.646	0.30	1.38
Skeletal muscle mass index (kg/m^2^)	0.700	2.01	0.78	5.16	0.145

Dependent variable: RYGB.

**Table 5:** Comparison of the studied variables in the group submitted to RYGB with and without vitamin D deficiency (*n*=49).

Variable	25(OH)D < 20 *μ*g/dL (*n*=27)	25(OH)D ≥ 20 *μ*g/dL (*n*=22)	*p* value
*Studied variables*			
Age (years)	45.0 ± 9.6	45.0 ± 8.4	0.994
Ethnicity (white)	18 (66.7%)	17 (77.3%)	0.530
Menopause (yes)	11 (40.7%)	8 (36.4%)	0.777
Use of contraception (yes)	1 (3.7%)	5 (22.7%)	0.077
Smoking (yes)	0 (0.0%)	3 (13.6%)	0.084
Supplementation (yes)	17 (63.0%)	16 (72.7%)	0.549
Regular photoprotection (yes)	10 (37.0%)	10 (45.5%)	0.574
Time since surgery (years)	3.5 ± 1.2	3.2 ± 0.9	0.361
BMI variation (kg/m^2^)	32.4 ± 4.8	28.0 ± 3.3	0.002
25(OH)D (ng/mL)	14.2 ± 3.5	32.9 ± 10.0	<0.001
Parathormone (pg/mL)	69.9 ± 25.2	52.2 ± 23.3	0.015
*Body composition*			
Current BMI (kg/m^2^)	32.6 ± 4.2	30.1 ± 4.5	0.149
Total muscle mass (kg)	47.9 ± 5.3	46.7 ± 4.9	0.417
Total skeletal muscle mass (kg)	18.4 ± 4.9	17.2 ± 6.8	0.507
Skeletal muscle mass index (kg/m^2^)	7.7 ± 1.2	7.5 ± 1.3	0.655
Adipose mass (kg)	31.8 ± 8.7	27.7 ± 7.2	0.096
Total fat percentage (%)	38.2 ± 5.8	35 ± 6.0	0.129

BMI: body mass index; significance level of Student's *t*-test.
